# Listening to tuna: Acoustic characterization of captive juvenile yellowfin tuna (*Thunnus albacares*)

**DOI:** 10.1111/jfb.70507

**Published:** 2026-06-30

**Authors:** Regi Fiji Anggawangsa, Frédéric Bertucci, Ignatius Tri Hargiyatno, Jhon Harianto Hutapea, Ananto Setiadi, Gunawan Gunawan, Wudianto Wudianto, Fayakun Satria, Marc Soria, Laurent Dagorn, Manuela Capello

**Affiliations:** ^1^ MARBEC, Université de Montpellier, CNRS, Ifremer, IRD Sète France; ^2^ National Research and Innovation Agency Jakarta Indonesia

**Keywords:** acoustic signature, bioacoustics, passive acoustic monitoring (PAM), sound production, *Thunnus albacares*, tropical tuna

## Abstract

The effectiveness of passive acoustic monitoring (PAM) for studying marine biodiversity highly relies on comprehensive libraries of species‐specific sounds. While sound production is well‐documented in reef and freshwater fishes, the acoustic behaviour of ecologically and economically vital pelagic species like tunas still remains largely unexplored. This study provides a detailed characterization of sounds produced by the yellowfin tuna (*Thunnus albacares*), a widely distributed species sustaining a global fishery. Acoustic recordings were conducted in a large concrete tank to minimize acoustic artefacts and ensure species‐specific attribution, where 19 juveniles were maintained in captivity during 16 days. Over 6400 sounds were detected and categorized into two distinct types: short sounds (average duration 47 ms) and long sounds (534.4 ms) consisting of trains of pulses, both with a peak frequency of 263.1 ± 31.3 Hz. There was a clear diel difference in yellowfin tuna sound production: long sounds were more abundant during the day than at night, whereas short sounds varied more modestly, peaking in the evening and declining overnight, with intermediate levels during the day. This characterization is a critical first step towards using PAM for monitoring this tropical tuna species, thereby providing a novel, fisheries‐independent method to study its distribution, behaviour and abundance.

## 1 INTRODUCTION

Sound production is currently documented in nearly a thousand species of ray‐finned fishes (Actinopterygii) across 133 families, and therefore represents a widespread form of communication rather than a mere by‐product of third‐party activities such as feeding or swimming. Even elasmobranchs (sharks, rays and skates), which were long considered silent, have recently been reported as active sound producers thanks to a few species of rays (Almagro & Barría, 2024; Barroil et al., 2024; Fetterplace et al., 2022) and sharks (Nieder et al., 2025). These sounds are generated through mechanisms such as stridulation, swim bladder vibrations or jaw movements (Parmentier & Fine, 2016; Rice et al., 2022). This diversity of sound‐producing mechanisms illustrates repeated evolutionary innovations, often co‐opting anatomical structures originally adapted for other functions (Parmentier & Fine, 2016; Parmentier & Lecchini, 2022), resulting in a large diversity of sound types. In marine and freshwater environments, fishes produce sounds in a wide range of social and ecological contexts, including territorial defence, competition for food, predator avoidance, prey detection, courtship, mate choice and the coordination of gamete release (Ladich & Fay, 2013; Parmentier & Fine, 2016). Furthermore, while acoustic signals could also act as species‐specific cues, their discriminability appears to be higher at the genus or family levels, due to greater divergence in sound‐producing mechanisms (Banse et al., 2024).

Over the past decade, passive acoustic monitoring (PAM) has rapidly expanded as a non‐invasive technique, using autonomous recorders to assess fish biodiversity and investigate specific ecological and behavioural processes through the detection and classification of sounds (Lindseth & Lobel, 2018; Mooney et al., 2020; Rountree et al., 2006). For example, acoustic recordings have been used to identify spawning sites and seasons (Jublier et al., 2020; Picciulin et al., 2020; Rowell et al., 2012), to document diel and seasonal rhythms of fish activity and to describe the succession of acoustic communities across habitats and time (Boyle et al., 2022; Ruppé et al., 2015).

To fully interpret the ecological patterns revealed by in situ PAM, it is essential to know which species are responsible for the recorded signals. Without this link, patterns of activity, community structure or spawning events remain difficult to interpret. For this reason, in situ surveys are often complemented by controlled recordings of individual fishes in aquariums or tanks, which provide reference libraries of species‐specific sounds. These targeted datasets are crucial for matching field recordings to their biological sources and thereby enhancing the ecological value of PAM. Additionally, these recordings in captivity make it possible to describe acoustic signals finely, characterize their temporal and spectral structure and identify species‐specific signatures with a high degree of confidence, which is essential to build sound libraries and to train automated classification tools (Ibrahim et al., 2018; Looby et al., 2023). Therefore, once described, sounds may fully serve as natural markers of species presence and behaviour and can also be used as proxies to estimate biodiversity (Mooney et al., 2020; Parsons et al., 2022).

Although research on sound production in reef‐associated and freshwater fishes is relatively advanced—supported by evidence that around half of coral reef fish families include at least one soniferous species (Parmentier et al., 2021; Rice et al., 2022)—many sounds remain undescribed, underscoring the need for comprehensive cataloguing (Darras et al., 2025; Desiderà et al., 2019; Di Iorio et al., 2021; Puebla‐Aparicio et al., 2024), particularly in the pelagic realm, where acoustic behaviour remains poorly documented. Early work by Marshall (1967) had, however, hypothesised on the basis of anatomical data, that sound production might likely be widespread among deep‐sea fish, a conclusion that can be extended to the entire pelagic environment. As Rountree et al. (2012) subsequently pointed out, empirical data have remained scarce since Marshall's hypothesis, mainly due to the inaccessibility of these environments.

Addressing this knowledge gap is particularly important, as PAM can provide insights into habitats where visual surveys are challenging, such as in the open ocean. This is especially true for highly migratory open‐ocean taxa such as the Scombridae, a family that includes tunas, mackerels and bonitos.

Tunas (*Thunnus* spp.) are among the most ecologically and economically important marine species, sustaining global fisheries worth approximately USD 40 billion annually (FAO, 2024). As apex predators, they play a crucial role in pelagic ecosystems, regulating prey populations and maintaining food web stability (Heidrich et al., 2023; Juan‐Jordá et al., 2011). Despite their economic and ecological values, fundamental aspects of tuna biology, including acoustic communication, remain poorly understood. Past research has focused on their vision (Kawamura et al., 1981a, 1981b; Matsuura et al., 2010; Nakamura, 1968) and lateral line perception (Ghysen et al., 2010), while the potential role of sound production remains underexplored. Pioneering studies have indicated that some tuna species are capable of sound production, potentially during feeding or social interactions (Allen & Demer, 2003; Fish, 1954; Fish et al., 1952; Fish & Mowbray, 1970). However, the full range of sounds produced by tuna, along with their acoustic characteristics, has yet to be thoroughly investigated. Sound production has been documented in species such as frigate tuna (*Auxis thazard*), Atlantic mackerel (*Scomber scombrus*), Atlantic bluefin tuna (*Thunnus thynnus*), yellowfin tuna (*Thunnus albacares*) and skipjack tuna (*Katsuwonus pelamis*), with sounds likely produced through hydrodynamic movement or muscular mechanisms (Allen & Demer, 2003; Fish, 1954; Fish et al., 1952; Fish & Mowbray, 1970; Miyake, 1952). Yet, detailed analyses on sound characteristics, including frequency ranges, temporal patterns and behavioural contexts, remain scarce. Most recordings have been made in noisy environments and with other species, making sound characterization and species assignment difficult. Furthermore, studying captive tunas remains challenging due to the difficulties in capturing and transporting individuals without compromising their condition, as well as the logistical demands of maintaining them in captivity, given their need for large spaces and tanks capable of holding substantial volumes of water.

The present study aims to investigate sound production in yellowfin tuna (*T. albacares*) by characterizing the acoustic properties and temporal occurrence of sounds emitted during intraspecific interactions and communication in captive individuals. Yellowfin tuna is a highly productive, fast‐growing and widely distributed species in tropical and subtropical areas (Block & Stevens, 2001; Collete et al., 2021; Collette & Nauen, 1983; Garcia, 1995). It accounts for nearly 30% of global tuna catches, and most stocks are currently at sustainable levels (FAO, 2024; ISSF, 2024). However, fishing pressure and climate‐driven habitat shifts pose significant pressures on this species, necessitating accurate monitoring to regularly assess its health status (Collete et al., 2021; Erauskin‐Extramiana et al., 2023). Currently, the available knowledge on the spatial distribution and abundance of yellowfin tuna is primarily derived from fisheries‐dependent data, which contain gaps due to limited fisheries coverage and potential biases introduced by management measures (such as quotas that influence catch data) or fishing gear selectivity. Moreover, substantial knowledge gaps persist regarding the biology, ecology and behaviour of yellowfin tuna. Establishing a reference for their sound characteristics can provide a basis for the application of PAM of yellowfin tuna in the wild, thereby facilitating the study of their behaviour and contributing to a global understanding of their spatial distribution and abundance, as well as enhancing the broader characterization of acoustic diversity of pelagic ecosystems.

## 2 MATERIALS AND METHODS

### 2.1 Captive tuna

Juveniles of yellowfin tuna (*T. albacares*) (fork length, i.e. from the tip of the snout to the posterior end of the middle caudal rays; range: 33–53 cm, Table S1) were collected during multiple fishing expeditions conducted between August and September 2023 using handlines deployed near fish aggregating devices (FADs) in the coastal waters off Gondol, Bali, Indonesia (8.156°S, 114.715°E). Following capture, individuals were immediately transferred to a specially designed live transport tank installed on the fishing boat, which maintained continuous seawater circulation and supplemental aeration to ensure optimal water quality during the approximately 1 to 2‐h transit to the BRIN Gondol research facilities. Upon arrival, fish were initially housed in a 4 m diameter, 1 m depth circular medical tank for 1–2 days of acclimatization, during which they received antibiotic treatment to prevent potential infections. Following this quarantine period, specimens were gently introduced into a large cylindrical concrete tank measuring 8 m in diameter and 3 m in depth. This tank, situated within a semi‐open‐air building, was provided with natural daylight while ensuring environmental protection. A continuous flow of seawater sourced directly from the nearby coast was maintained and supplemented with aeration. Fish received a daily morning feeding (8:00 AM–9:00 AM) consisting of small pelagic fish. Throughout the holding period, feeding responses and general health were closely monitored.

**TABLE 1:** Summary of the main acoustic characteristics of yellowfin tuna short (*n* = 60) and long (*n* = 26) sounds (average ± standard deviation).

Metrics	Short sounds	Long sounds
Pulse peak frequency (Hz)	263.1 ± 31.3	261.5 ± 21.5
Pulse duration (ms)	19.1 ± 6.8	17.5 ± 4.5
Pulse period (ms)	23.4 ± 7.7	31.1 ± 9.2
Sound duration (ms)	47 ± 13.5	534.4 ± 232.1
Number of pulses	2.1 ± 0.7	17.6 ± 8.7

During the study period, the number of tunas in the tank ranged between 9 and 18 individuals (see Table S1). This study was approved by the National Research and Innovation Agency of Indonesia (BRIN) with ethical clearance approval Ref No: 098/KE.02/SK/12/2022.

### 2.2 Acoustic recordings

An autonomous acoustic recorder (SNAP, Loggerhead Instruments, Sarasota, FL, USA) connected to an HTI‐96‐min hydrophone (sensitivity: 170 dB re 1 V μPa‐1; flat frequency response range: 2 Hz–30 kHz; High Tech Inc., Long Beach, MS, USA) was mounted on a custom weighted support and positioned vertically on the floor near the centre of the tank, with the hydrophone oriented upwards. The system was programmed to record 1 min every 5 min at a sampling frequency of 44.1 kHz with a 16‐bit resolution, from 2 to 17 September 2023. Control recordings were first conducted from 14 to 21 July 2023, to characterize ambient noise conditions before fish introduction.

### 2.3 Data processing

**FIGURE 1:**
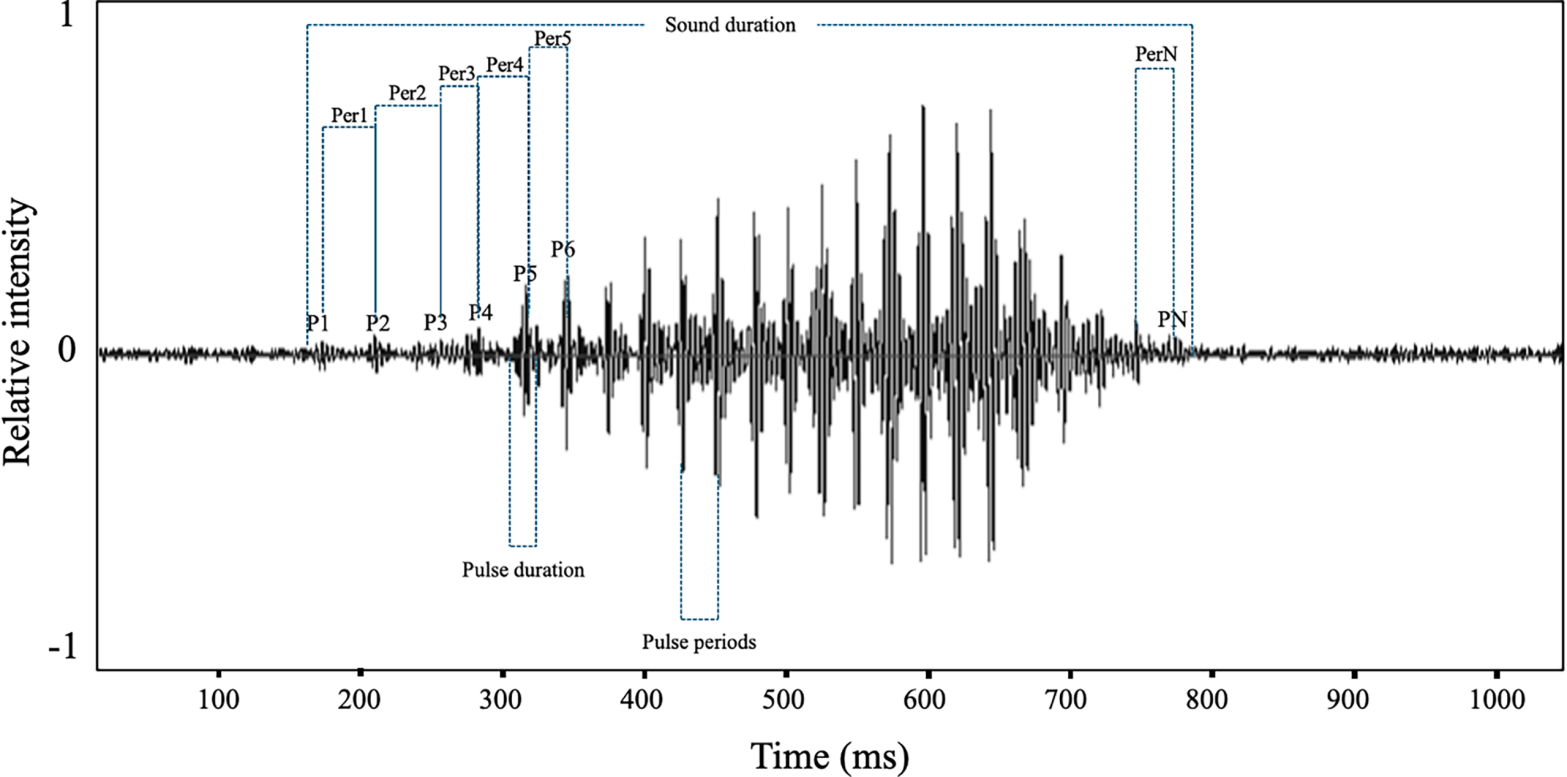
Oscillogram of a sound produced by *Thunnus albacares* and illustration of the temporal acoustic features measured: sound duration, pulse duration, pulse period, number of pulses (PN) and period number (Per1 to PerN).

Acoustic data were analysed using Raven Pro 1.6.5 (Cornell Lab of Ornithology, USA). A bandpass filter (150–2500 Hz) was applied to remove low‐frequency noise from the pump engine and high‐frequency noise generated by water circulation and aerators, while also focusing on the most biologically relevant frequency range for fish hearing and vocalization (Lobel et al., 2010; Tavolga, 2012). Recordings were screened aurally and visually to identify sounds, and high‐quality sounds with good signal‐to‐noise ratio were selected for detailed analysis. Sounds were classified into two categories based on their duration and number of pulses. Sounds with a duration of less than 100 ms and fewer than 3 pulses were categorized as ‘short sounds’, while the remaining sounds were categorized as ‘long sounds’. For each sound type, temporal features were measured from oscillograms (intensity vs. time representation of a signal) and consisted in the total sound duration (from the beginning of the first pulse to the end of the last one, ms), the number of pulses, the pulse duration (ms) and the pulse period (peak‐to‐peak interval between two successive pulses, ms) (Figure 1). Pulse peak frequency (Hz) was measured from spectrograms (frequency vs. time representation of a signal) by selecting a pulse (Figure S1) and using the *Peak Frequency measurement* feature in Raven [512‐point Fast Fourier Transform (FFT) with a Hamming window]. The spectral composition of the sounds was characterized using power spectral density (PSD) estimates, calculated with the seewave package in R (Sueur et al., 2008). To characterize the temporal and intensity patterns of long sounds, the pulse period (ms) and relative intensity (measured as the ratio of the measured intensity to the maximum intensity) were analysed as a function of their sequential order.

### 2.4 Statistical analysis

Because individual sound sources could not be distinguished, statistical analyses were conducted using the total acoustic output of all individuals in the tank. All sound characteristics (sound duration, pulse duration, pulse periods, number of pulses and pulse peak frequency) were tested for normality using the Shapiro–Wilk test. The Shapiro–Wilk test indicated that none of the sound characteristics followed a normal distribution (*W* = 0.62–0.66, all *p*‐values <10^−3^). Mann‐Whitney *U* tests were then performed to compare sound characteristics between the two sound types (short vs. long sounds).

For each sound type, the detected sounds were aggregated into hourly counts to examine diel patterns in acoustic activity. These hourly counts were subsequently divided into four distinct time periods of 6 h each, for comparative analysis: night (12:00 AM–5:59 AM); morning (6:00 AM–11:59 AM); afternoon (12:00 PM–5:59 PM); and evening (6:00 PM–11:59 PM). A Kruskal–Wallis test was first conducted to compare the number of sounds between these different periods. If a significant difference was detected, post‐hoc pairwise comparisons using Wilcoxon rank‐sum tests with the Hochberg adjustment method were performed. Finally, for long sounds, the Mann–Kendall test was conducted to identify temporal patterns in pulse period duration and intensity. To this purpose, the test was used to determine if the measured values exhibited a statistically significant monotonic trend across the sequence of pulse numbers. All tests were performed with R Studio (Version 2022.02.3), and the significance level *α* = 0.05 was used.

## 3 RESULTS

### 3.1 Sound types characteristics

**FIGURE 2:**
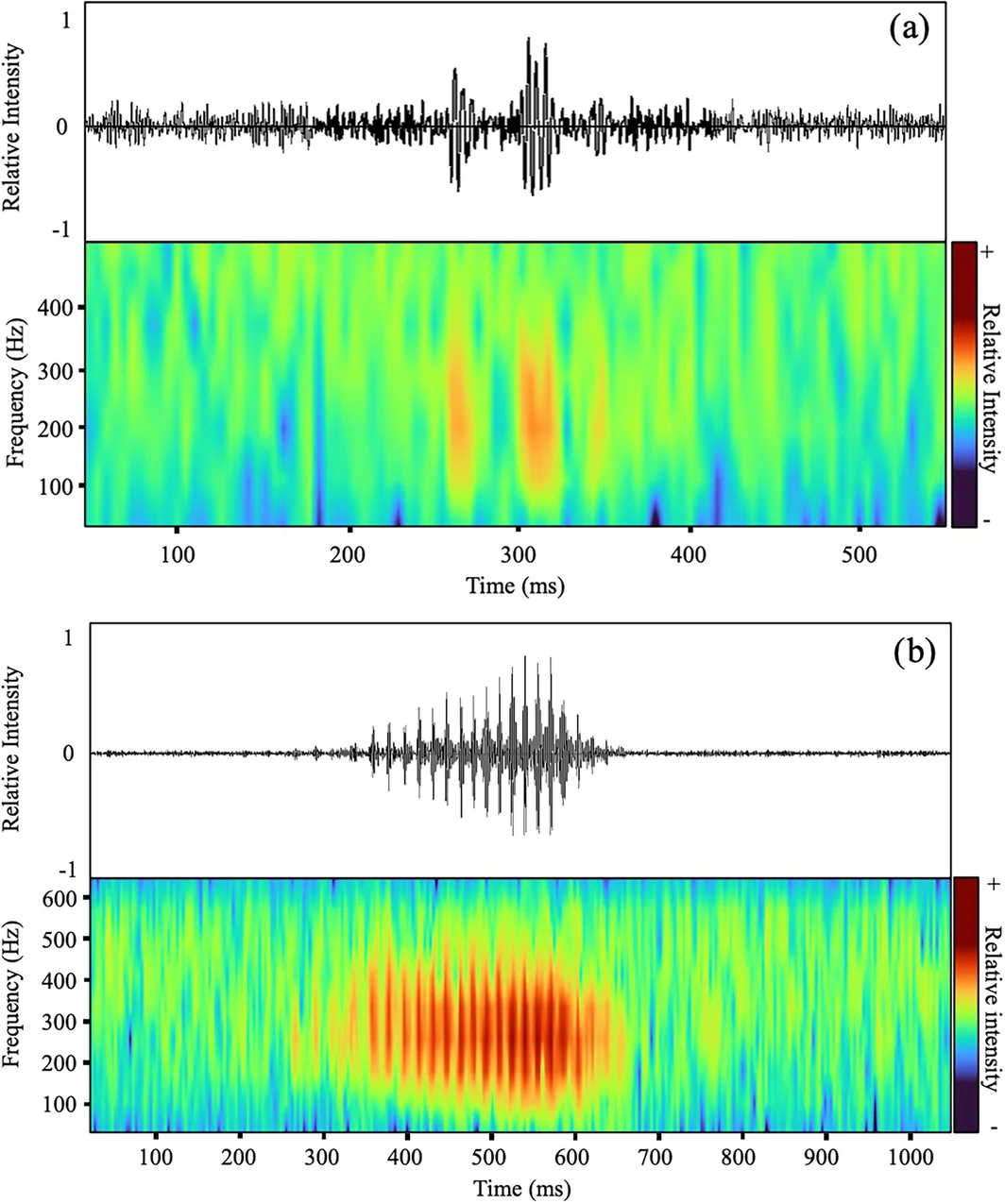
Two sound types produced by *Thunnus albacares*: (a) Oscillogram (top panel) and spectrogram (bottom panel) of a short sound. (b) Oscillogram (top panel) and spectrogram (bottom panel) of a long sound. The colour scale on the right indicates relative intensity, where warmer colours represent higher acoustic energy. Spectrogram parameters: Fast Fourier Transform (FFT) size = 512 points, Hamming window, 50% overlap. Audio files illustrating each sound type can be found in the Supporting Information.

A total of 6412 sounds were detected and categorized into short sounds (*n* = 5905) and long sounds (*n* = 507) (Figure 2) throughout the recordings with tunas in the tank. In contrast, neither short nor long sounds were identified during the control study. Among the detected sounds, 60 short sounds and 26 long sounds with good signal‐to‐noise ratios were randomly selected for further analysis. The short sounds (Figure 2a, Table 1) exhibited a mean ± SD duration of 47 ± 13.5 ms, ranging from 19.6 to 77.9 ms. This sound type consisted of 1 to 3 pulses per sound, with a pulse peak frequency of 263.1 ± 31.3 Hz. The pulse duration was 19.1 ± 6.8 ms, ranging from 8.4 to 28.1 ms, and the pulse period was 23.4 ± 7.7 ms, ranging from 10.4 to 46.2 ms. The long sounds (Figure 2b, Table 1) had an average duration of 534.4 ± 232.1 ms, ranging from 163.6 to 872.1 ms. These sounds consisted of 17.6 ± 8.7 pulses, ranging from 4 to 34 pulses. The pulse period was 31.1 ± 9.2 ms, ranging from 12.8 to 71.9 ms, while the pulse duration was 17.5 ± 4.5 ms, ranging from 8.6 to 46.9 ms. The pulse peak frequency for long sounds was 261.5 ± 21.5 Hz. The power spectra for both sound types showed energy was concentrated in the lower portion of the frequency range, that is, 150–350 Hz, which was consistent with the identified pulse frequency ranges (172.3–344.5 Hz), and with amplitude then declining progressively towards higher frequencies (Figure S2).

**FIGURE 3:**
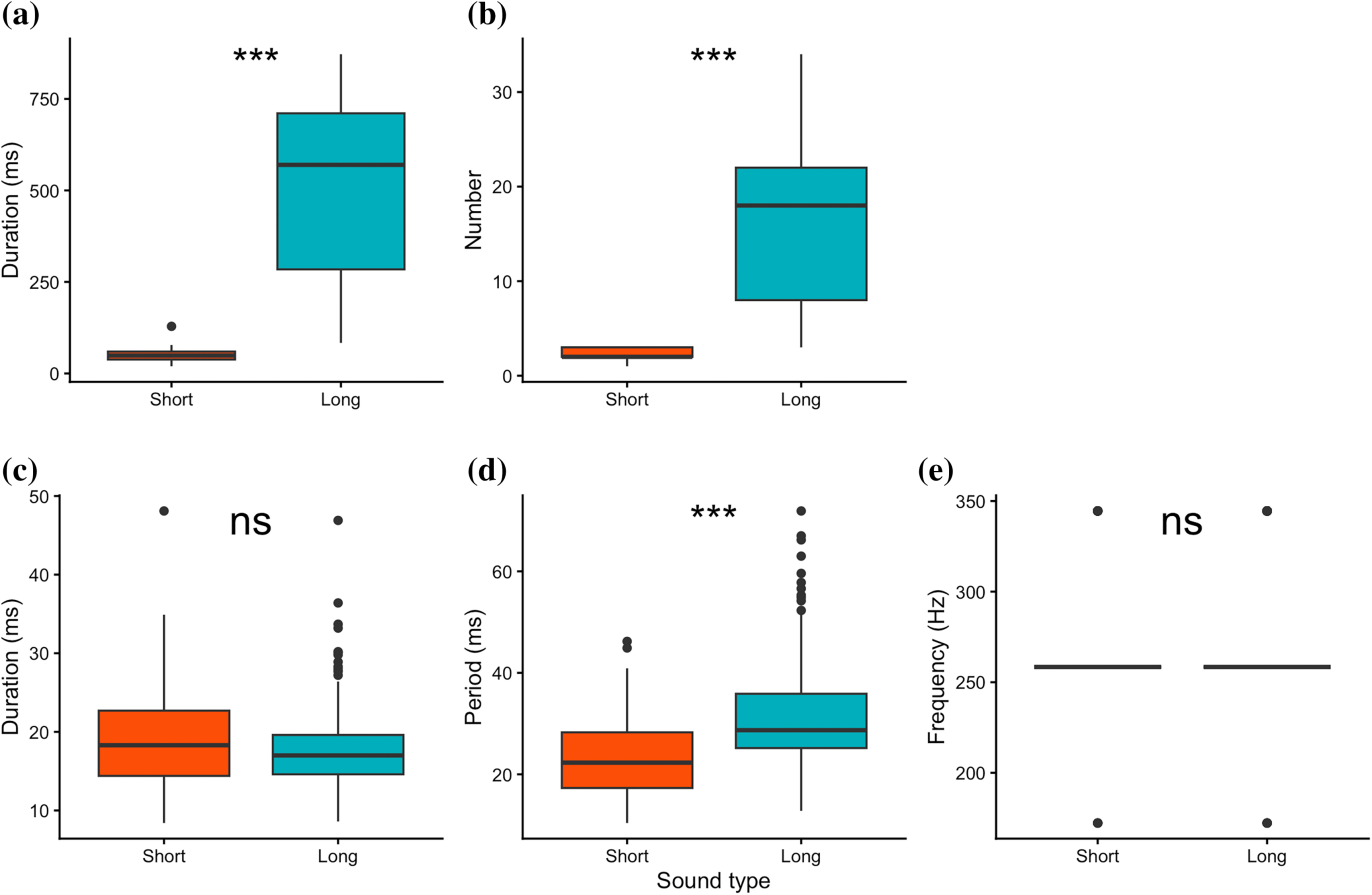
Comparison of acoustic characteristics between short (red) and long (blue) sound types in *Thunnus albacares*: (a) sound duration, (b) number of pulses, (c) pulse duration, (d) pulse period (ms) and (e) peak frequency of pulses (Hz). The boxplot elements show median (centre line), interquartile range (box), 1.5× interquartile range intervals (whiskers) and outliers (dots). Significance levels: ns (not significant), *p < 0.05, **p < 0.01, ***p < 0.001.

The Mann–Whitney *U* test showed significant differences in sound duration (*W* = 1, *p* < 10^−3^), pulse period (*W* = 6437, *p* < 10^−3^) and number of pulses (*W* = 9, *p* < 10^−3^) between the two sound types. No significant differences were found for pulse duration (*W* = 16,475, *p* = 0.097) or pulse peak frequency (*W* = 28,818, *p* = 0.47) (Figure 3).

**FIGURE 4:**
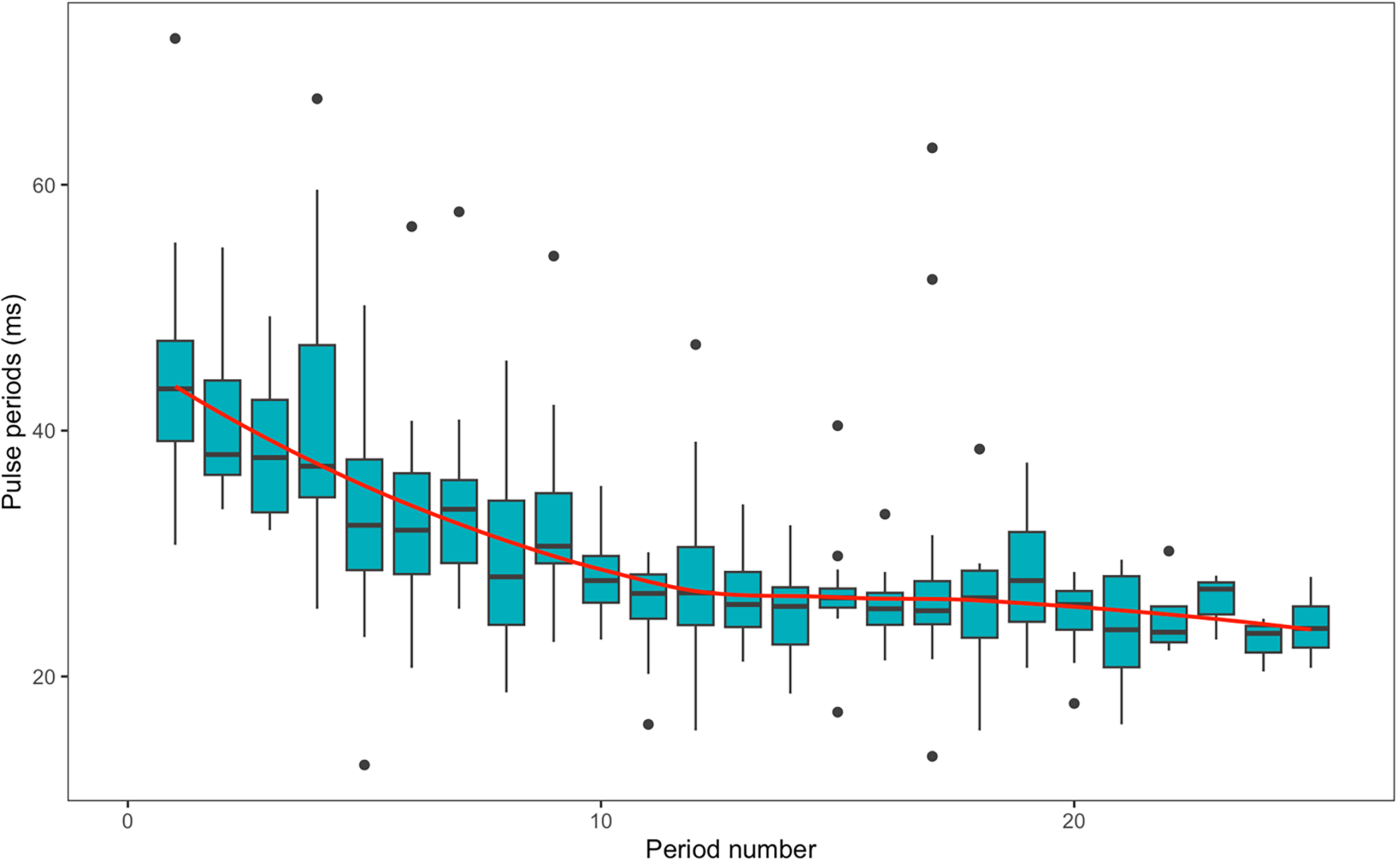
Pattern of pulse periods of a long sound as a function of period number. The boxplot elements are the same as in Figure 3; red line: smoothed trend line (loess method).

**FIGURE 5:**
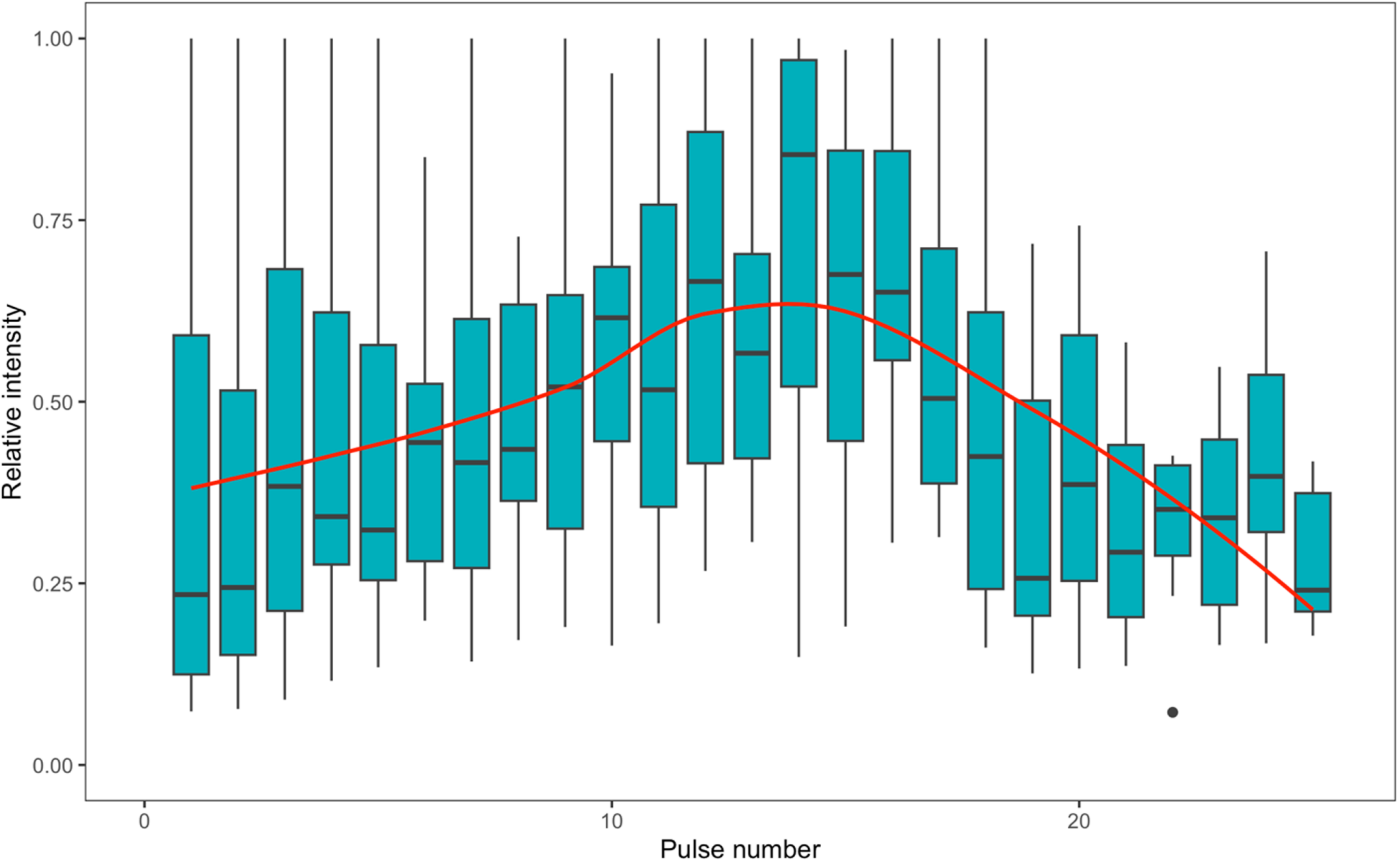
Pattern of relative intensity (calculated as the proportion of the maximum intensity measured) of a long sound as a function of pulse number. The boxplot elements are the same as in Figure 3; red line: smoothed trend line (loess method).

Long sounds—particularly those with a high (>15) number of pulses—exhibited a distinct temporal pattern in their pulse period duration (Kendall test, *τ* = −0.52, *p*‐value <10^−3^). These sounds began with a pulse period of 44.1 ± 9.9 ms, which significantly decreased until period number 10 to 28.2 ± 3.5 ms, and then remained stable around 26.3 ± 5.8 ms (Figure 4). A temporal pattern was also observed in the relative intensity of pulses, with pulse intensity increasing from the first pulse until pulse number 14 (Kendall test, *τ* = 0.29, *p*‐value <10^−3^), and then gradually decreasing in longer sounds until the sound ended (Kendall test, *τ* = −0.51, *p*‐value <10^−3^) (Figure 5).

### 3.2 Diel acoustic activity

**FIGURE 6:**
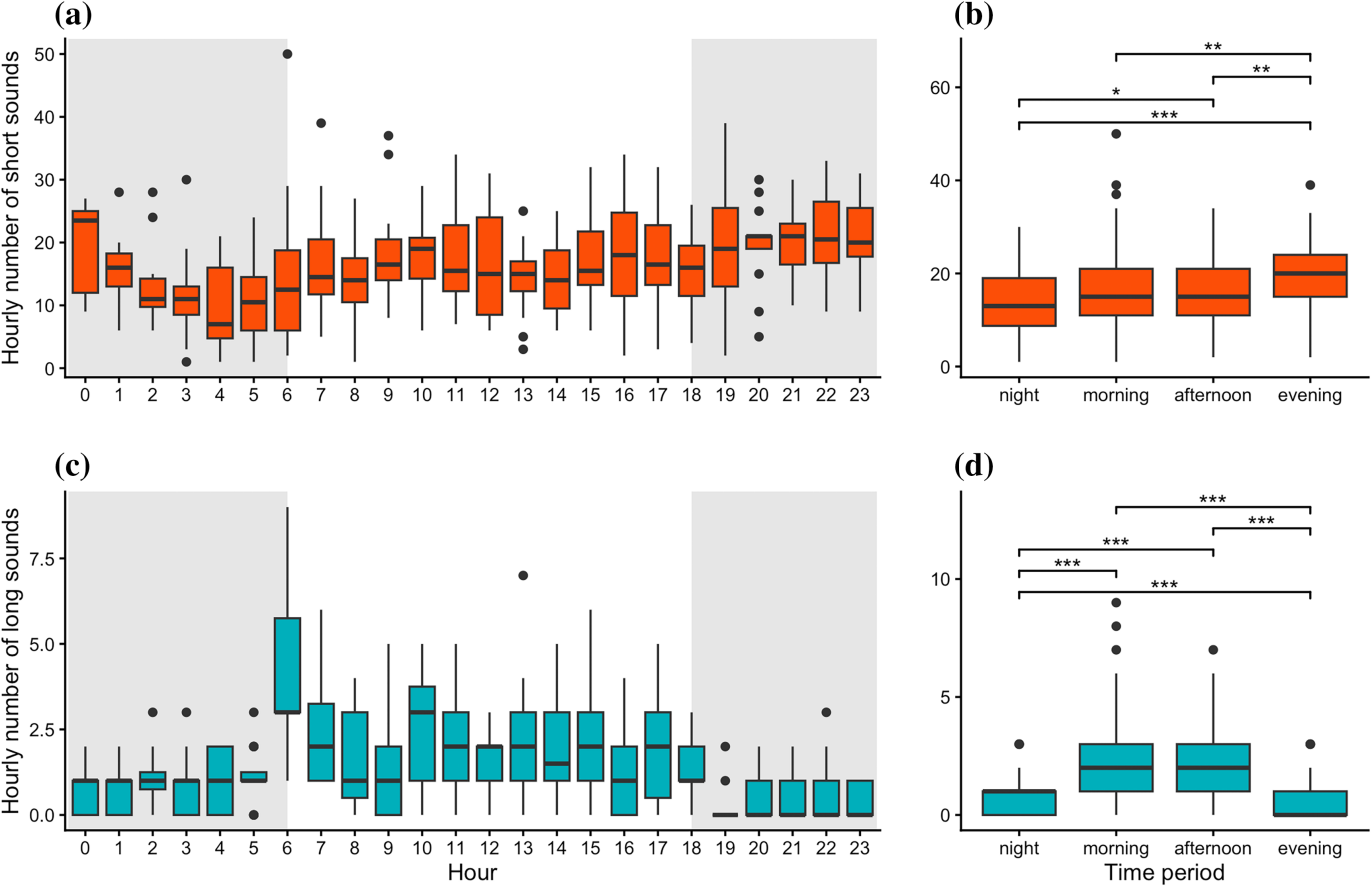
Daily pattern of short sound (red) and long sound (blue) detections. (a) and (c): Hourly number of sounds (white background represents daytime and grey background represents night‐time); (b) and (d): hourly number of sounds per 6‐h time period (morning: 6:00 AM–11:59 AM; afternoon: 12:00 PM–5:59 PM; evening: 6:00 PM–11:59 PM; night: 12:00 AM–5:59 AM). The boxplot elements and significant levels are the same as in Figure 3.

A significant difference was observed in the number of short sounds recorded across the four time periods (Kruskal–Wallis *χ* ^2^ = 29.67, df = 3, *p*‐value <10^−3^). The production of short sounds was highest during the evening (6:00 PM–11:59 PM) and lowest during the morning (6:00 AM–11:59 AM). However, the morning period was non‐significantly different from both afternoon and night (Figure 6a, Table S2).

Significant differences were also found in the number of long sounds detected (Kruskal–Wallis *χ* ^2^ = 79.66, df = 3, *p*‐value <10^−3^). Yellowfin tuna exhibited the highest production of long sounds during daytime hours (6:00 AM–5:59 PM) than in the evening (6:00 PM–11:59 PM, when the number of sounds was the lowest) and at night (12:00 AM–5:59 AM) (Figure 6, Table S2). It is noticeable that long sounds are almost absent at the start (5:00 AM) and end (7:00 PM) of the day.

## 4 DISCUSSION

Aquatic environments, in particular coastal waters, can be rich acoustic landscapes where sounds provide essential information for orientation and communication for a large variety of organisms, and for ecological monitoring. While sound production in reef and freshwater fish is now well documented, the acoustic behaviour of pelagic species remains much less studied. This gap is all the more important to fill given the potential of passive acoustics to reveal biodiversity and monitor population dynamics in remote environments where direct observation is difficult, such as the open ocean. In this context, the present study provides new insights into the sound‐producing capabilities of an emblematic pelagic species.

Previous research has indicated that this species might be vocal, but it also emphasized the importance of conducting recordings in dedicated, single‐species experimental tanks (Allen & Demer, 2003). Although recording wild animals in their natural habitat is generally preferred over studying captive individuals (Banse et al., 2023; Duncan et al., 2016; Rogers et al., 2016), using tanks ensures that only the sounds produced by the species of interest are recorded. Moreover, the large experimental tanks used in this study, along with the extended monitoring period, facilitated the observation of captive individuals acclimatized to their new environment. Finally, the use of large concrete tanks minimized the risk of altered sound properties and acoustic artefacts, which are more likely to occur in smaller tanks made of glass, plastic or fibreglass (Akamatsu et al., 2002; Banse et al., 2023; Okumura et al., 2002; Parmentier et al., 2014).

Our study revealed that when captive juvenile yellowfin tuna individuals were present in the tank, two distinct sound types could be identified, that is, ‘short’ and ‘long’ sounds, while none of these sounds were found during the control experiment, with no fish present in the tanks. This latter observation rules out external or environmental sources, providing robust evidence that the acoustic signals characterized in this study are indeed produced by the tuna themselves. The short sounds showed a degree of similarity to those previously documented in captive yellowfin tuna by Allen and Demer (2003); however, our findings revealed substantial discrepancies in acoustic parameters. Specifically, the sounds recorded in the present study were notably shorter in duration (47 ± 13.5 ms) compared to ca. 100 ms recorded by Allen and Demer (2003) and exhibited a substantially higher pulse frequency (263.1 ± 31.3 Hz compared to 20–130 Hz). These pronounced differences are likely attributable to both methodological and biological factors, including better recording quality in the current study that minimized the high levels of background noise which may have masked finer acoustic details in the earlier work, as well as a difference in the size of the specimens observed (33–53 cm *L*_f_ here vs. larger 50–100 cm *L*_f_ individuals in the previous study). Furthermore, the acoustic environment in Allen and Demer's experiment was more complex, involving an aquarium community that included other species such as bluefin tuna, barracuda, bat rays and sea turtles, which could have influenced the recorded sounds. While an earlier study by Miyake (1952) also reported comparable short‐duration sounds in captive yellowfin tuna, it did not provide a detailed spectral and temporal analysis of their acoustic properties. Furthermore, this study was also conducted in mixed‐species tanks and pond environments, alongside other tuna species and several small reef fishes. In contrast, the present study is the first to document the long sounds of yellowfin tuna.

While sharing a consistent fundamental frequency range of 172.27 to 344.53 Hz, short and long sounds exhibited significant differences in their temporal structure and pulse characteristics. The fundamental frequencies of these sounds align with the known hearing sensitivity of tuna. According to Finneran et al. (2000), which relies on previous findings from Iversen (1967), yellowfin tuna are most sensitive to frequencies between 200 and 800 Hz. While our findings demonstrate that sounds produced by juvenile tuna fall within this range, it is acknowledged that tuna sounds are likely size‐dependent. As indicated by Allen and Demer (2003), larger tuna individuals can produce dominant frequencies below 130 Hz, suggesting that both sound production and hearing sensitivity may shift as the fish grows.

Determining whether a sound is accidental or communicative requires considering both its acoustic structure and its behavioural context. Accidental sounds of hydrodynamic origin (e.g. tail flicks, collisions, feeding strikes) are typically irregular, broadband and lack consistent temporal or spectral patterns (Kasumyan, 2008; Ladich & Fine, 2006). In contrast, communicative sounds in fishes generally have stereotyped features: they are often composed of repeated pulses or pulse trains with characteristic durations, intervals and frequency ranges that remain stable within a species (Amorim, 2006; Parmentier & Fine, 2016). In this respect, both sound types identified in this study, characterized by well‐defined frequencies and structures, are likely produced for communication purposes. Furthermore, communicative sounds are usually associated with specific social contexts—such as courtship, territorial defence or agonistic encounters—where they can be reliably produced and recognized by conspecifics (Amorim et al., 2015). In our study, which involved only juvenile individuals, reproductive behaviour can be excluded; however, the presence of multiple individuals in the tank, combined with the well‐known social behaviour of tunas (Freon & Dagorn, 2000), supports the hypothesis that these sounds may be linked to social interactions, including communication between individuals to facilitate schooling or swarming. Although, recording yellowfin tuna sounds in tanks may not fully represent their natural behaviour, given their fast swimming and extensive diel vertical and horizontal movements (Bone & Moore, 2008; Collette & Nauen, 1983).

In this study, long sounds were more abundant during the day, raising the possibility that they are associated with specific behaviours. In the wild, tuna schools tend to be more loosely aggregated at night than during the day (Josse & Bertrand, 2000; Trygonis et al., 2016), which could provide a context for such diel variation. However, one might instead expect the opposite pattern, with increased communication at night, so the underlying drivers remain unclear. For short sounds, 24‐h differences were present but relatively modest, with a noticeable increase in the evening and lower production towards the end of the night. This may also reflect changes in activity, yet linking these patterns to behaviours observed in natural settings, whether schooling dynamics or aggregation around FADs, is challenging given the highly specific, confined conditions of the present study. The near‐absence (or very low occurrence) of long sounds around 5:00 AM and 7:00 PM is intriguing. It remains unclear whether this reflects individual‐specific fluctuations in activity within the 19 recorded fish, or instead points to an endogenous rhythm in sound production. Further studies will be required to disentangle these possibilities. In contrast, short sounds did not exhibit a comparable pattern, except maybe around 8:00 PM. Future research on captive and wild individuals, combining acoustic triangulation with video footage, could help capture the behavioural context of sound production and clarify the functional significance of these sounds.

While the mechanism of sound production in yellowfin tuna (*T. albacares*) remains unclear, potential mechanisms can be inferred. First, the fact that short and long sounds occur at nearly the same frequencies suggests that they are produced by the same sound‐producing organ(s). In numerous teleost fish, sound is produced through the rapid contraction of specialized sonic muscles often associated with the swim bladder (Fine & Parmentier, 2015; Parmentier et al., 2017). Allen and Demer (2003) previously posited that such mechanisms, that is, swim bladder muscle contractions, could be responsible for sound production in this species. Certain observed acoustic temporal patterns, such as the progressive shortening of pulse periods in long sounds, align with this hypothesis. However, this interpretation should be approached with caution, because the present study was limited to juveniles within a relatively narrow size range (33–53 cm *L*_f_), which inherently restricts the observed variability in sound characteristics and precludes extrapolation to adult populations. Furthermore, it remains uncertain whether the sonic musculature, even if this mechanism is accurate, is fully developed and functional at the juvenile stage. Consequently, while a swim bladder‐based mechanism remains plausible, future studies should include detailed morphological investigations to exclude alternative possibilities. This research must encompass a broader size and age spectrum.

The detailed characterization of *T. albacares* sounds conducted on captive individuals provides a foundational dataset for detecting its presence in the wild. While future research in the wild should also focus on validating the present laboratory‐derived acoustic characterization in natural environments, this study establishes the essential spectral and temporal parameters required to identify this species during PAM‐based surveys, as well as to develop automatic detection algorithms. These tools would enable a non‐invasive monitoring of tuna populations through passive acoustic methods, offering an alternative to traditional population assessment techniques that rely on fisheries‐dependent data. A key behavioural trait of this species, its strong tendency to associate with floating objects (Freon & Dagorn, 2000), represents a unique opportunity to apply this technology effectively. This behaviour is shared by other tropical tunas—such as skipjack and bigeye tuna—along with various pelagic fish species, including dolphinfish, rainbow runner and certain shark species. Because FADs, floating structures deployed by tuna fishers, already form an extensive and widely distributed array across remote oceanic regions, they offer a ready‐to‐use platform for developing PAM in environments that are otherwise difficult to access (Moreno et al., 2016). Recently, novel abundance indices for tropical tunas that exploit their associative behaviour with FADs have been developed (Baidai et al., 2024). Equipping FADs with hydrophones could provide an unprecedented opportunity to complement these indices and implement PAM for tuna monitoring. Such an approach would not only contribute to the assessment of *T. albacares* distribution and abundance but would also provide an additional tool for studying the behaviour and ecology of this species, including unresolved questions regarding the role of social interactions in tuna associative dynamics (Capello et al., 2022). This knowledge is key to assessing the impacts of FAD fisheries on tuna populations (Capello et al., 2023; Dagorn et al., 2013; Dupaix et al., 2024). Furthermore, equipping FADs with hydrophones could provide valuable insights into the broader and largely unexplored pelagic biodiversity associated with these structures.

## AUTHOR CONTRIBUTIONS

**R.F.A.:** Conceptualization; data curation; formal analysis; methodology; visualization; writing—original draft; writing—review and editing. **F.B.:** Conceptualization; data curation; formal analysis; methodology; visualization; validation; supervision; writing—review and editing. **I.T.H.:** Writing—review and editing. **J.H.H.:** Supervision; writing—review and editing. **A.S.:** Writing—review and editing. **G.G.:** Writing—review and editing. **W.W.:** Project administration; supervision; writing—review and editing. **F.S.:** Project administration; supervision; validation; writing—review; and editing. **M.S.:** Supervision; writing—review and editing. **L.D.:** Conceptualization; funding acquisition; project administration; supervision; validation; writing—review; and editing. **M.C.:** Conceptualization; data curation; methodology; funding acquisition; project administration; supervision; formal analysis; validation; writing—review and editing. All authors reviewed a first draft and approved the submission.

## CONFLICT OF INTEREST STATEMENT

The authors declare no conflicts of interest.

## Supporting information


**Table S1.** Timeline showing the number of yellowfin tuna individuals monitored each day in the experimental tank and their fork length (*L*_f_) statistics (min, max; mean and standard deviation) throughout the acoustic recording period. Since there were no mortalities or transfers after 12 September 2023, the last line indicates data from that date until the end of the experiment.
**Table S2.** Pairwise comparisons of acoustic activities among groups of 6‐h time periods (morning: 6:00 AM–11:59 AM; afternoon: 12:00 PM–5:59 PM; evening: 6:00 PM–11:59 PM; night: 12:00 AM–5.59 AM) for short and long sound types. *p*‐Values were calculated using the Wilcoxon rank‐sum test and adjusted for multiple comparisons using the Post Hoc Hochberg method. Significance levels: ns (not significant), *p < 0.05, **p < 0.01, ***p < 0.001.
**Figure S1.** Spectrogram of a sound produced by *Thunnus albacares* and illustration of how the box for pulse peak frequency measurement was made in Raven.
**Figure S2.** Power spectral density (PSD) of captive juvenile yellowfin tuna (*Thunnus albacares*) sounds. Averaged power spectra were calculated using Welch's method for long sound (black), short sound (blue) and background noise (grey). Spectral analysis was performed using a 2048 point FFT, Hamming Windows, 50% overlap.

**File S1:** Example of long sound type of juvenile yellowfin tuna (*Thunnus albacares*), recorded at BRIN Gondol, Bali, 11 September 2023.

**File S2:** Example of short sound type of juvenile yellowfin tuna (*Thunnus albacares*), recorded at BRIN Gondol, Bali, 12 September 2023.

## Data Availability

The data that support the findings of this study are available from the corresponding author upon reasonable request.
