# Supplementary material for: Listening to tuna: Acoustic characterization of captive juvenile yellowfin tuna (Thunnus albacares)
Source: J Fish Biol. 2026 Jun 30;109(3):1725–36. doi: 10.1111/jfb.70507 (PMC13602105; doi:10.1111/jfb.70507)
Supplement: Supplementary file 1 [file JFB-109-1725-s002.docx]

**SUPPLEMENTARY**

**TABLE S1** Timeline showing the number of yellowfin tuna individuals monitored each day in the experimental tank and their fork length (*L_f_*) statistics (min, max; mean and standard deviation) throughout the acoustic recording period. Since there were no mortalities or transfers after 12/09/2023, the last line indicates data from that date until the end of the experiment.

| **Date** | **Number of**  **individuals** | **Size (*L_f_*, cm)** | |  |
| --- | --- | --- | --- | --- |
|  |  | **Min-Max** | **Mean +/- SD** | |
| 02/09/2023 to 05/09/2023 | 9 | 48-53 | 51.3 ± 1.5 | |
| 06/09/2023 | 10 | 35-53 | 49.8 ± 5.2 | |
| 07/09/2023 | 10 | 35-53 | 49.8 ± 5.2 | |
| 08/09/2023 | 10 | 35-53 | 49.8 ± 5.2 | |
| 09/09/2023 | 19 | 33-53 | 42.4 ± 8.7 | |
| 10/09/2023 | 19 | 33-53 | 42.4 ± 8.7 | |
| 11/09/2023 | 18 | 33-53 | 42.9 ± 8.7 | |
| 12/09/2023 to 17/09/2023 | 18 | 33-53 | 42.9 ± 8.7 | |

**TABLE S2** Pairwise comparisons of acoustic activities among groups of 6-hour time periods (morning: 6.00-11.59 h; afternoon: 12.00-17.59 h; evening: 18.00-23.59 h; night: 00.00-05.59 h) for short and long sound types. P-values were calculated using the Wilcoxon rank-sum test and adjusted for multiple comparisons using the Post Hoc Hochberg method. Significance levels: ns (not significant), * p < 0.05, ** p < 0.01, *** p < 0.001

| **Group 1** | **Group 2** | **Adjusted p-value** | **Significance**  **level** |
| --- | --- | --- | --- |
| **Short sounds** | | | |
| night | morning | 0.072 | ns |
| night | afternoon | 0.041 | * |
| night | evening | < 0.001 | *** |
| morning | afternoon | 0.800 | ns |
| morning | evening | 0.008 | ** |
| afternoon | evening | 0.011 | ** |
| **Long sounds** | | | |
| night | morning | < 0.001 | *** |
| night | afternoon | < 0.001 | *** |
| night | evening | 0.002 | *** |
| morning | afternoon | 0.120 | ns |
| morning | evening | < 0.001 | *** |
| afternoon | evening | < 0.001 | *** |

**Figure S1** Spectrogram of a sound produced by *T. albacares* and illustration of how the box for pulse peak frequency measurement was made in Raven.

**Figure S2** Power spectral density (PSD) of captive juvenile yellowfin tuna (*Thunnus albacares*) sounds. Averaged power spectra were calculated using Welch’s method for long sound (black), short sound (blue), and background noise (grey). Spectral analysis was performed using a 2048 point FFT, Hamming Windows, 50% overlap.
